# Coagulation factor stability and sterility of thawed fresh frozen plasma stored at 2-6 ^o^ C for five days: Towards optimizing utilization

**DOI:** 10.1016/j.htct.2025.103988

**Published:** 2025-10-08

**Authors:** Ruchika Bhartia, Paramjit Kaur, Kshitija Mittal, Anita Tahlan, Varsha Gupta, Ravneet Kaur, Gagandeep Kaur

**Affiliations:** Government Medical College and Hospital, Chandigarh, India

**Keywords:** Fresh Frozen Plasma, Thawed plasma, Blood coagulation factor

## Abstract

**Background:**

Fresh frozen plasma plays a crucial role in managing trauma and bleeding patients. The concern about a decline in labile coagulation factors limits its usage beyond 24 hours. This study aimed to analyze coagulation factor levels and microbial contamination of thawed fresh frozen plasma stored at 2-6 °C for five days.

**Material and methods:**

A prospective observational study was conducted on 40 male donors with blood groups A and O selected through purposive sampling. Blood was collected in 450 mL bags and freshly prepared plasma was aliquoted and frozen at -80 °C. Aliquots were thawed at 37 °C and tested on Days 0, 1, and 5 after storage at 2-6 °C. Coagulation screening assays and activity of coagulation factors V, VIII, IX, fibrinogen, and von Willebrand factor were performed. Samples were tested for sterility on Day 5.

**Results:**

One-way ANOVA revealed a significant increase in mean prothrombin time, activated partial thromboplastin time, and international normalized ratio during storage (p-value < 0.001). The activity of factors V and VIII showed a significant decrease over five days (factor V - 20.0 % and factor VIII - 42.2 %; p-value < 0.001), with factor VIII activity declining by 30.8 % within the first 24 hours and remaining relatively stable thereafter. Mean von Willebrand factor activity was lower in fresh frozen plasma from O blood group donors (p-value < 0.05) on Days 1 and 5 of storage using an unpaired t-test. Cultures were sterile on Day 5.

**Conclusion:**

Key coagulation factors were well preserved in thawed plasma till five days of storage at 2-6 °C without compromising product sterility suggesting potential for extended shelf life.

## Introduction

Fresh frozen plasma (FFP) is a rich source of coagulation factors. It is currently indicated for transfusion in conditions of impaired hemostasis resulting from multiple coagulation factor deficiencies such as liver diseases, disseminated intravascular coagulation, major trauma, and as a replacement fluid during therapeutic plasma exchange [1,2]. FFP should be thawed at 30-37 °C in a plasma thawing bath before transfusion. As per the Indian National Regulatory Authority, FFP should be used within 24 hours of thawing [3]. If not utilized within 24 hours, FFP is discarded due to concerns about a decline in coagulation factor activity and microbial contamination. This leads to the wastage of this precious resource.

The shelf life of thawed FFP is a topic of interest. The recommendations pertaining to the maximum permitted storage time post-thawing vary in different countries. The Association for the Advancement of Blood and Biotherapies (AABB) introduced the concept of thawed plasma (TP) to maintain inventory and reduce plasma wastage. TP is plasma stored at 1-6 °C for an additional four days after the 24 hours period [4]. The British Society of Hematology states that once thawed, FFP may be stored at 2-6 °C for up to 24 hours [5]. In Australia, TP is known as Extended Life Plasma (ELP) and has a shelf life of up to five days at 2-6 °C from the day of thawing. TP and ELP are similar to FFP but have lower levels of labile coagulation factors [6, 7, 8, 9]. In developing countries like Malaysia, FFP is discarded if unused within six hours of thawing [10].

In severe trauma-induced bleeding, precious lives can be saved by rapid replacement of coagulation factors with the administration of plasma. Availability of pre-thawed FFP in trauma centers will be a great asset to clinicians for immediate resuscitation of these patients. Therefore, the present study aimed to determine the levels of labile and stable coagulation factors and to check for bacterial contamination of thawed plasma during storage at 2-6 °C for five days. Utilization of FFP beyond 24 hours of thawing may help in timely availability and prevent potential wastage of unused thawed FFP.

## Materials and methods

### Study design

This prospective cross-sectional study was conducted in the Department of Transfusion Medicine of a tertiary care hospital. This study was conducted on 40 healthy male donors over 12 months after approval from the Institutional Ethics Committee and informed written consent from blood donors. All whole blood donors were screened as per the Indian National Regulatory Authority [3]. Blood donors meeting the eligibility criteria and weighing more than 55 kg were included in the study using a purposive sampling method. Only male donors were included as female donors usually do not meet the minimum weight eligibility criteria of 55 kg for the collection of 450 mL blood. Exclusion criteria were donors where blood was not collected using a single clean venipuncture, collection time of more than eight minutes, technical errors, under or over-collected bags, and donors reactive for any transfusion-transmitted infections. The donors were further divided into one of two groups:

Group 1 (n = 20): donors of the O Rh D positive blood group and
Group 2 (n = 20) donors of the A Rh D positive blood group.

### Blood collection and processing

After obtaining informed consent, whole blood was collected in 450 mL triple blood bags with Citrate Phosphate Dextrose Adenine Solution (CPDA-1) as an anticoagulant (Terumo Penpol, Thiruvananthapuram, India). Whole blood was collected within eight minutes using a single clean venipuncture. Packed red blood cells (PRBCs), FFP, and platelet concentrates (PCs) were prepared within six hours of blood collection according to the departmental standard operating procedure using the same cryofuge by a dedicated trained laboratory technologist. Plasma was rapidly frozen at -80 °C in a mechanical deep freezer (Terumo Penpol, Thiruvananthapuram, India) immediately after preparation. Once frozen, FFP was subsequently stored below –30 °C in a deep freezer (Terumo Penpol, Thiruvananthapuram, India).

FFP units were thawed in a plasma thawing bath (Helmer Scientific, Indiana, USA) at 37 °C for 30 minutes within three months of freezing. After thawing, 50 mL aliquots were prepared in a transfer bag using a sterile connecting device (TSCD-II, Terumo Penpol, Thiruvananthapuram, India), and a baseline 10 mL sample was taken in a plain tube after proper mixing of contents. The aliquot was stored at 2-6 °C and sampled again at 24 hours and five days for coagulation factor analysis. The rest of the unit was stored at 2-6 °C for issue to patients within 24 hours.

### Laboratory analysis

Coagulation screening assays, including Prothrombin time (PT), Activated Partial thromboplastin time (APTT), Prothrombin index (PTI), and International normalized ratio (INR), were analyzed on Days 0, 1, and 5 using a fully automated coagulation analyzer (ACL Top TM 500 CTS, Instrumentation Laboratory, Bedford, MA, USA) as per the manufacturer’s instructions. Factor V (FV), factor VIII (FVIII), factor IX (FIX), fibrinogen and von Willebrand factor (vWF) levels were also measured using the same analyzer as per the manufacturer’s instructions. The thawed FFP samples were also sent for sterility testing on Day 5.

### Statistical analysis

The sample size was estimated based on the mean difference of FVIII activity in thawed FFP based on a previous study [10]. For possible attrition, it was decided to include at least 30 subjects. The following formula was used for sample size calculation n = (Zα/2+Zβ)2 *2*σ2 / d2 where Zα/2 is the critical value of the normal distribution at α/2 (e.g. for a 95 % confidence level, α is 0.05 and the critical value is 1.96), Zβ is the critical value of the normal distribution at β (e.g. for a power of 95 %, β is 0.05 and the critical value is 1.64), σ2 is the population variance, and d is the difference between means.

All data was compiled using Google Sheets and data analysis was done using IBM SPSS statistics software. For quantitative variables, data were analyzed either as the mean and standard deviation or as the median and interquartile range. The distribution of the variables was tested with the chi-square test. Student t-test was applied to compare two groups and the ANOVA test was applied to compare three groups for normally distributed data. Categorical variables were reported as counts and percentages. Discrete (categorical) groups were compared by the chi-square (χ^2^) test. The ANOVA test was applied for time-related variables of scores/skewed data; the paired t-test was carried out for normally distributed data. Comparisons of categorical data were made using the Pearson chi-square test. All the statistical tests were two-sided and were performed at a significance level of α = 0.05, thus a p-value < 0.05 was considered significant.

## Results

The mean age of the study population was 34.3 ± 9.3 years with a range of 19-60 years. The mean time taken to collect 450 mL of whole blood from all the study subjects was 6.2 ± 0.83 minutes (range: 5-8 minutes). The mean time between whole blood collection and component preparation was 3.7 ± 0.88 hours (range: 2-5 hours). The mean time between the day of collection and thawing was 73.3 ± 19.6 days (range: 35-103 days).

### Conventional coagulation tests of thawed fresh frozen plasma

Table 1 shows the changes over five days of storage at 2-6 °C in the values of conventional coagulation tests of thawed FFP. The baseline PT ranged between 10.2 s and 14.1 s while the APTT ranged between 26.1 s and 37.2 s. According to one-way ANOVA, the mean increases in PT and APTT values were statistically significant over five days of storage (p-value < 0.001). The increases in mean PT of thawed FFP stored at 2-6 °C were statistically significant from Day 0 to Day 1 (p-value = 0.004), Day 0 to Day 5 (p-value < 0.001), and Day 1 to Day 5 (p-value < 0.001) using an unpaired t-test. The changes in mean APTT from Day 0 to Day 1 (p-value < 0.001), Day 0 to Day 5 (p-value < 0.001), and Day 1 to Day 5 (p-value < 0.05) were also statistically significant using an unpaired t-test.

**Table 1:** Conventional coagulation tests of thawed fresh frozen plasma over five days of storage at 2-6 °C.

Parameter	Normal Range	Day 0 (Mean ± SD)	Day 1 (Mean ± SD)	Day 5 (Mean ± SD)	p-value ANOVA
**Prothrombin Time (s)**	9.4-12.5	11.3 ± 0.9	11.9 ± 0.9	13.0 ± 1.2	**<0.001**
**PTI (%)**	96-100	107.0 ± 11.0	98.7 ± 10.1	87.3 ± 10.6	**<0.001**
**APTT (s)**	25.1-36.5	30.9 ± 2.7	33.1 ± 3.1	34.6 ± 3.2	**<0.001**
**International Normalized Ratio**	0.9-1.1	0.94 ± 0.1	0.99 ± 0.1	1.1 ± 0.1	**<0.001**

PTI: prothrombin time index; APTT: activated partial thromboplastin time; SD: Standard Deviation.

Note: Bold p-values indicate statistically significant results.

### Coagulation factors in thawed fresh frozen plasma

The activities of FV, FVIII, FIX, and vWF, and fibrinogen levels in thawed FFP over five days of storage at 2-6 °C are shown in Table 2. The baseline FV activity ranged between 73.5 % and 137.5 %, and FVIII activity ranged between 43 % and 184.8 %. The coagulation FV and FVIII activities in thawed FFP showed significant declines during storage at 2-6 °C over five days using the one-way ANOVA test (p-value < 0.001; Table 2). The decline in FVIII activity from Day 0 to Day 5 was 42.2 % with a major decline (30.8 %) in activity being observed in the first 24 hours. Out of 40 donors, 55 % (n = 22) had FVIII activity greater than 50 % while in 20 % (n = 8) of donors the activity was more than 70 % on Day 5. The FIX activity, vWF activity and fibrinogen levels remained stable over the five-day storage period of thawed FFP at 2-6 °C. The mean activities of all the factors studied were well within the reference ranges on Day 5 of storage at 2-6 °C.

**Table 2:** Coagulation factors in thawed fresh frozen plasma over five days of storage at 2-6 °C.

Parameter	Normal range	Day 0 (Mean ± SD)	Day 1 (Mean ± SD)	Day 5 (Mean ± SD)	p-value*	Reduction percentage on Day 1	Reduction percentage on Day 5
**Factor V activity (%)**	62-139	98.7 ± 16.6	93.1 ± 23.3	78.7 ± 17.4	**<0.001**	5.6	20.0
**Factor VIII activity (%)**	50-150	97.7 ± 30.4	66.9 ± 24.1	55.5 ± 19.1	**<0.001**	30.8	42.2
**Factor IX activity (%)**	65-150	117.1 ± 18.6	109.6 ± 22.5	111.5 ± 20.2	0.248	7.5	5.6
**Fibrinogen (mg/dl)**	238-498	257.0 ± 54.8	272.3 ± 79.3	317.4 ± 53.4	**<0.001**	**-**	**-**
**von Willebrand Factor (%)**	Blood group O: 41.1-125.9 Blood group A: 61.3-157.8	112.5 ± 40.5	111.2 ± 37.2	102.6 ± 35.5	0.456	1.3	9.9

*Using one-way ANOVA test

SD: Standard Deviation

Note: Bold p-values indicate statistically significant results.

### Donor blood group and coagulation parameters in thawed fresh frozen plasma

The mean FVIII activity was lower in FFP from donors of the O blood group as compared to A blood group donors on all three days; however, the difference was statistically insignificant. Mean FIX activity was significantly lower in FFP from O blood group donors on Day 5 of storage (p-value: 0.029). Mean vWF activity was also lower in FFP from O blood group donors with the difference being significant on Days 1 (p-value = 0.014) and 5 of storage (p-value = 0.019). The mean fibrinogen levels were significantly higher in FFP prepared from O blood group donors on Day 1 of storage (p-value: < 0.001; Table 3).

**Table 3:** Donor blood group and coagulation parameters of thawed fresh frozen plasma.

Days of storage	Blood group ‘O’	Blood group ‘A’	p-value**
**Factor V (%)**			
Day 0	100.4 ± 18.2	96.9 ± 15.1	0.518
Day 1	96.1 ± 26.5	89.9 ± 19.5	0.413
Day 5	77.6 ± 17.9	79.9 ± 17.3	0.685
p-value*	**0.003**	**0.014**	
**Factor VIII (%)**			
Day 0	90.3 ± 32.2	105.4 ± 27.1	0.123
Day 1	61.7 ± 25.3	72.5 ± 22.1	0.165
Day 5	50.6 ± 16.3	60.7 ± 20.6	0.097
p-value*	**<0.001**	**<0.001**	
**Factor IX (%)**			
Day 0	112.5 ± 17.2	122.1 ± 19.3	0.109
Day 1	106.5 ± 22.3	112.8 ± 22.8	0.389
Day 5	104.7 ± 19.5	118.6 ± 18.9	**0.029**
p-value*	0.432	0.372	
**Fibrinogen (mg/dL)**			
Day 0	259.1 ± 47.2	254.9 ± 63.1	0.815
Day 1	375.3 ± 73.9	269.1 ± 86.4	**<0.001**
Day 5	321.3 ± 43.9	313.4 ± 62.8	0.650
p-value*	**<0.001**	**0.006**	
**von Willebrand factor (%)**			
Day 0	100.7 ± 35.6	125.1 ± 42.4	0.058
Day 1	97.3 ± 34.8	125.9 ± 34.7	**0.014**
Day 5	89.9 ± 34.5	116.0 ± 32.2	**0.019**
p-value*	0.610	0.655	

* Using one-way ANOVA test; ** Using unpaired t-test; Note: Bold p-values indicate statistically significant results.

### Culture of thawed fresh frozen plasma

All thawed FFP samples were sent for culture on Day 5 of storage. The cultures were sterile after 72 hours of incubation.

## Discussion

Trauma accounts for nearly 20 % of mortality in India, with a high impact on the younger population [11]. FFP is crucial for managing trauma-related coagulopathy, and pre-thawed liquid plasma could aid early resuscitation. However, guidelines for using thawed FFP beyond 24 hours at 2-6 °C are lacking in developing nations. Coagulation factors and sterility in thawed FFP from O and A Rh D-positive donors stored at 2-6 °C for five days were compared due to potential ABO influence on vWF and FVIII [8,12,13]. To ensure uniformity, FFPs were thawed within three months of storage.

In this study, the mean PT, PTI, APTT, and INR of thawed FFP increased significantly during storage likely due to coagulation factor deterioration at 2-6 °C. Nevertheless, all values remained within the normal reference range over five days. A study observed a significant increase in mean PT from 11.3 ± 0.83 s on Day 0 to 13.3 ± 1.10 s on Day 5 in thawed FFP stored at 2-6 °C [14]. Another study also showed 23 % increase in mean PT and APTT values from Day 0 to Day 5 of storage [15]. Authors have reported an increase in mean INR in thawed FFP from 0.95 ± 0.05 on Day 0 to 1.20 ± 0.21 on Day 5 of storage [8].

FV, a labile pro-coagulant factor with 40 % homology to FVIII, showed a 20 % activity loss during storage at 2-6 °C**.** However, the mean FV activity (78.7 ± 17.4 %) on Day 5 remained within the reference range and met the quality control criteria of 70 %, making it suitable for use in isolated FV deficiency. One study reported a decline (114.1 ± 38.7 % on Day 0 to 70.6 ± 18.9 % on Day 5) in mean FV activity in thawed FFP stored over five days at 1-6 °C [8].

FVIII activity declines during prolonged plasma storage at 2-6 °C and its measurement is a quality control requirement. In the present study, FVIII activity decreased by 43 % over five days, with a major drop (30.8 %) in the first 24 hours, with stabilization thereafter. This suggests that extending thawed FFP storage to five days does not adversely affect FVIII levels. Studies report a 36-60 % decline in mean FVIII activity by Day 5 in thawed FFP stored at 2-6 °C [15]. In this study, FVIII activity remained within the normal reference range up to five days of refrigerated storage, sufficient for achieving clinical hemostasis. Mean baseline FVIII activity was lower in group O donors, consistent with existing literature [12].

FIX showed a non-significant decrease in activity in thawed FFP throughout the storage period as seen in previous reports [8,14]. A significantly lower mean FIX activity was observed in FFP from group O donors on Day 5 of storage. A recent study from India also reported significantly lower FIX levels in O group donors as compared to non-O group donors [16].

Fibrinogen is a key protein involved in clot formation [17]. The current study found no significant decline in fibrinogen levels in thawed FFP over five days of refrigerated storage, consistent with previous reports [8,10,14,18]. This supports its use in hypofibrinogenemia, particularly in resource-limited settings. Fibrinogen levels in cryoprecipitate were higher in O group donors than A group donors on Day 1, challenging the notion that O group donors are unsuitable for cryoprecipitate due to lower FVIII and vWF levels. Given the high prevalence of the O group in India, further research is needed to explore its potential in cryoprecipitate production. vWF is a multifunctional protein and an acute-phase reactant [17]. In this study, vWF activity in thawed FFP remained stable during storage, whereas another study reported a decline from 110.8 ± 39.01 % at baseline to 67.0 ± 35.5 % on Day 5 [14]. A significantly lower vWF activity was found in O group donors on both Day 1 and Day 5, consistent with reports that vWF levels are 25 % lower in O group plasma due to the effect of ABO(H) carbohydrate structures on its glycans [19].

In the normal population, clotting factor levels range from 50-150 %, with hemostasis requiring much lower levels [7]. In this study, baseline coagulation factor activity remained above 70 %. Plasma is rarely contaminated due to frozen storage, but thawing in a water bath poses a risk. All samples remained sterile after 72 hours of incubation, supporting the safe storage of FFP for five days at 2-6 °C. Similarly, another study found no bacterial growth in culture samples collected on Day 5 and tested on Day 7 [20].

The present study had a few limitations. Firstly, it was a single-center study with a small sample size, which may limit the generalizability of the findings. The exclusion of female donors, primarily due to weight requirements, could have introduced gender-related bias by overlooking physiological differences. Larger, multi-center studies are needed to validate these results and assess clinical implications. Despite these limitations, the findings provide valuable insights for managing coagulopathies with FFP transfusions. Future research should explore the impact of extended storage on additional factors and assess potential cost-effectiveness.

## Conclusion

This study demonstrates that extending the storage duration of thawed FFP to five days at 2-6 °C is feasible while maintaining clinically effective levels of key coagulation factors and product sterility. This signifies the potential of pre-thawed FFP in improving patient care by facilitating faster resuscitation in trauma situations and reducing plasma wastage.

## Ethics approval statement

The study was approved by the institutional ethics committee and informed written consent was obtained from all participants.

## Funding information

None.

## Author contributions

**RB:** Conceptualization, Data curation, Formal analysis, Investigation, Methodology, Writing – original draft; **PK:** Conceptualization, Formal analysis, Methodology, Resources, Supervision, Validation, Writing – review & editing; **KM:** Formal analysis, Methodology, Supervision, Validation, Writing – review & editing; **AT:** Formal analysis, Methodology, Supervision, Validation; **VG:** Methodology, Supervision, Validation; **RK:** Methodology, Resources, Supervision, Writing – review & editing; **GK:** Methodology, Visualization, Writing – review & editing.

## Conflicts of interest

The manuscript has been seen and approved by all authors, it is not under active consideration for publication, has not been accepted for publication, nor has it been published, in full or in part.

## Data Availability

The data pertaining to the study is available from the first author and corresponding author upon reasonable request.
